# Clinical significance of sarcopenia in the treatment of patients with primary hepatic malignancies, a systematic review and meta-analysis

**DOI:** 10.18632/oncotarget.19687

**Published:** 2017-07-28

**Authors:** Guoqing Zhang, Songfeng Meng, Renfeng Li, Jianwen Ye, Longshuan Zhao

**Affiliations:** ^1^ Department of Hepatobiliary and Pancreatic Surgery, First Affiliated Hospital of Zhengzhou University, Zhengzhou, Henan Province, China

**Keywords:** sarcopenia, hepatocellular carcinoma, intrahepatic cholangio-carcinoma, third lumbar skeletal muscle index, third lumbar total psoas area

## Abstract

**Background:**

The impact of sarcopenia on outcomes following treatment for primary liver tumors remains contentious. Therefore, we performed a systematic literature review and meta-analysis to evaluate the clinical significance of sarcopenia in the treatment of patients with primary liver tumors.

**Data sources:**

A systematic literature search was performed in English through February 1, 2017 in databases.

**Results:**

There were significant differences between patients with and without sarcopenia in overall 1- and 3-year survival (1 year: OR: 0.43; 95% CI: 0.27-0.68; *P*=0.0004; 3 year: OR: 0.67; 95% CI: 0.47-0.96; *P*=0.03). However, overall 5-year survival showed no significant difference between the groups (OR: 0.61; 95% CI: 0.35-1.07; *P*=0.08). Patients with sarcopenia showed a significant 53% reduction in disease-free survival within 5 years (OR: 0.47; 95% CI: 0.28-0.79; *P*=0.005). Also, sarcopenia had a significantly negative impact on recurrence in patients with primary liver tumors (RR: 2.71; 95% CI: 1.46-5.05; *P*=0.002). Regarding complications rate, we concluded that there was a statistically significant difference between two groups in overall complications rate (RR: 2.52; 95% CI: 1.50-4.22; *P*=0.0005). However, the major complications rate showed no significant difference between the groups (RR: 1.19; 95% CI: 0.65-2.20; *P*=0.57).

**Conclusions:**

Sarcopenia seemed to have a negative effect on overall survival in patients with primary liver tumors in the early phase post-treatment, but further research is needed to investigate the prognostic impact on overall survival over the longer term. Moreover, sarcopenia could significantly increase the incidence rates of post-treatment recurrence and overall complications in patients with primary liver tumors.

## INTRODUCTION

Sarcopenia, which is defined as the loss of muscle mass and function [1], is critically involved not only in aging but also in a variety of chronic diseases, such as tuberculosis infection, chronic obstructive pulmonary disease (COPD), diabetes mellitus (DM), advanced organ failure and other wasting conditions. In recent years, studies have shown that sarcopenia is prevalent in patients with certain cancers [2, 3], and evidence has revealed its prognostic significance in oncologic patients [4–10]. A study by Chindaprasirt J [11] demonstrated that patients who suffered from sarcopenia had shorter median overall survival among cancer patients. More specifically, the frequency of sarcopenia varied greatly from 11.1% to 76% in patients with primary liver tumors (hepatocellular carcinoma (HCC) and intrahepatic cholangio-carcinoma (ICC)) [12–14], and the demonstration of the prognostic significance of sarcopenia has been reported relatively frequently in patients with primary liver tumors. To the best of our knowledge, several reviews [15–17] have shown that sarcopenia can be associated with impaired overall survival and increased postoperative morbidity in primary liver tumors. However, these studies have had several limitations. First, the reviews included studies evaluating different tumor types (including gastrointestinal and hepatopancreatobiliary malignancies). Second, the relationships between sarcopenia and the recurrence of malignancy or post-treatment complications (overall complications and major complications) were not described.

To date, it has been frustrating that there have been no systematic reviews specifically identifying sarcopenia in people with primary liver tumors, and the impact of sarcopenia on outcomes following treatment for primary liver tumors remains contentious. This systematic literature review and meta-analysis were performed to evaluate the clinical significance of sarcopenia in the treatment of patients with primary liver tumors. Outcomes of interest included overall survival, disease-free survival, recurrence of malignancy and post-treatment complications (overall complications and major complications).

## RESULTS

### Literature selection

**Figure 1:**
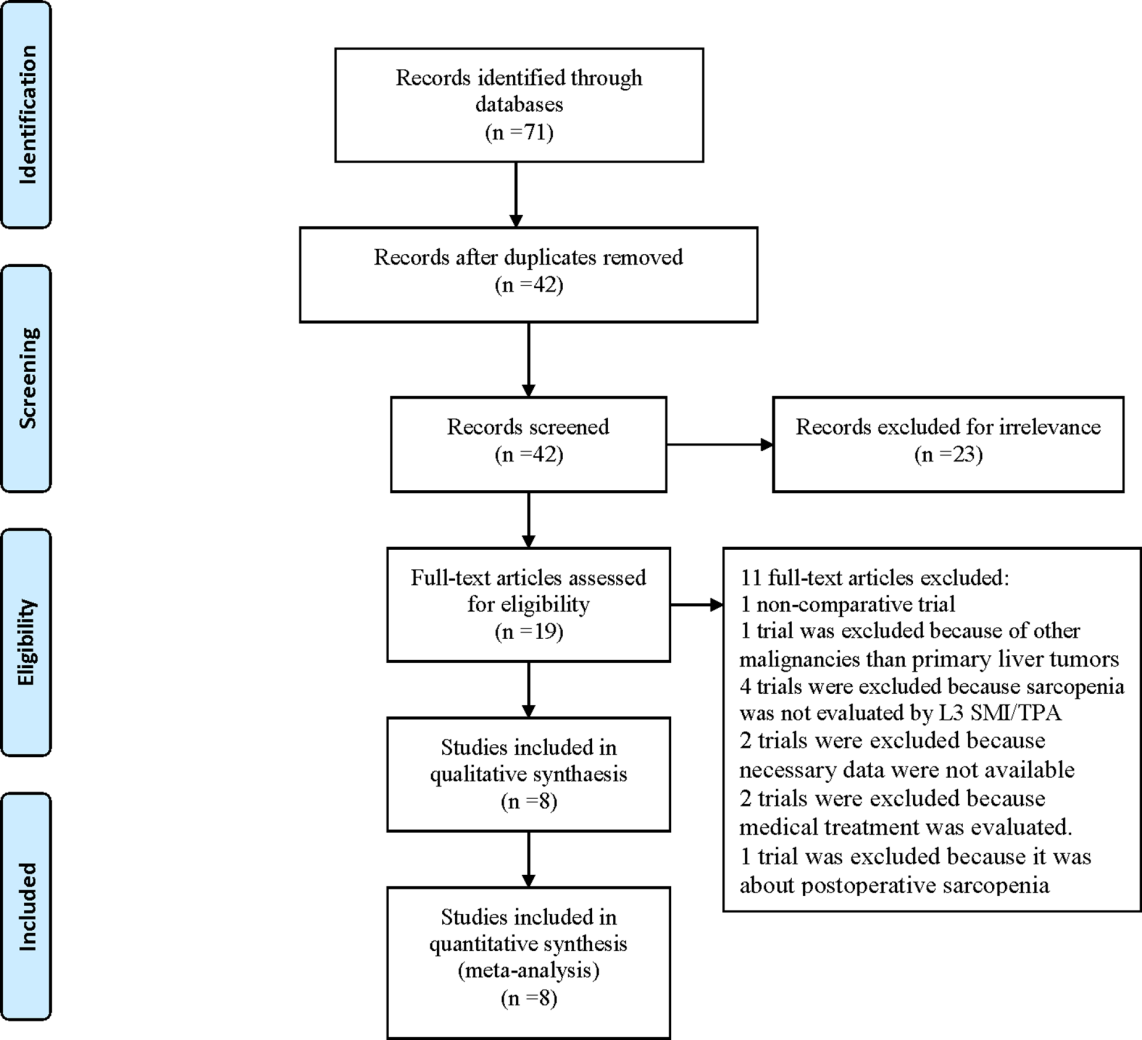
PRISMA 2009 Flow Diagram

Database searches yielded 71 entries, of which 52 were excluded because of duplications (29 trials) or irrelevance (23 trials). Of the 19 publications that qualified for abstract review, 1 was excluded because it was not a comparative trial, 1 trial was excluded because it included other malignancies than primary liver tumors, 4 trials were excluded because sarcopenia was not evaluated by the L3 skeletal muscle index (L3 SMI) or L3 total psoas area (L3 TPA), 2 trials were excluded because necessary data were not available, 2 trials were excluded because medical treatment was evaluated, and 1 trial was excluded because it was about postoperative sarcopenia. The Preferred Reporting Items for Systematic Reviews and Meta-Analysis (PRISMA) flow diagram for study selection is shown in Figure 1.

### Included trials and quality assessment

Detailed characteristics of the included patients are listed in Table 1. Of these 8 comparative trials, the Newcastle-Ottawa Scale (NOS) [18] was applied to determine the risk of bias in this literature. According to the scores (Table 2), all the included trials were considered high-quality (score ≥7) trials.

**Table 1:** The characteristics of the included studies

Study	country	Study design	Group assignment	Histopathological type	Treatment	CT scan	significant characteristics^^^	Definition of sarcopenia
**Harimoto et al 2013**	England	Retrospective	Sarcopenia	HCC	hepatectomy	performed preoperative	Sex, BMI, Albumin, ICGR15	L3 SMI ≤43.75 cm2/m2 for men and 41.10 cm2/m2 for women;
			No-sarcopenia					
**Levolger et al 2015**	USA	Retrospective	Sarcopenia	HCC	Hepatectomy or RFA	performed <3 months prior to or 3 days after treatment	BMI	L3 SMI ≤52.0 cm2/m2 for men and≤ 39.5 cm2/m2 for women;
			No-sarcopenia					
**Valero et al 2015**	USA	Retrospective	Sarcopenia	HCC or ICC	Resection or LT	performed <60 days prior to or 10 days after treatment	NR	L3 TPA ≤784.0 mm2/m2 for men and≤642.1 mm2/m2 for women;
			No-sarcopenia					
**Voron et al 2015**	USA	Retrospective	Sarcopenia	HCC	Hepatectomy	performed <2 months prior to or 7 days after treatment	Age, Stature, BMI, Albumin	L3 SMI ≤52.4 cm2/m2 for men and ≤38.9 cm2/m2 for women;
			No-sarcopenia					
**Harimoto et al 2016**	Netherlan ds	Retrospective	Sarcopenia	HCC	Hepatectomy	performed preoperative	HBV, HCV, Skeletal muscle mass	the actual L3 SMI was 85% smaller than the calculated skeletal muscle area^&^;
			No-sarcopenia					
**Kamachi et al 2016**	Netherlan ds	Retrospective	Sarcopenia	HCC	Hepatectomy or RFA	performed preoperative	Sex, BMI, DM, Albumin, PT	L3 SMI ≤52.4 cm2/m2 for men and 38.5 cm2/m2 for women;
			No-sarcopenia					
**Yabusaki et al 2016**	England	Retrospective	Sarcopenia	HCC	Hepatectomy	performed preoperative	Sex, BMI, Albumin, Number of tumors	L3 SMI ≤43.75 cm2/m2 for men and 41.10 cm2/m2 for women;
			No-sarcopenia					
**Takagi et al 2016**	Japan	Retrospective	Sarcopenia	HCC	Hepatectomy	performed <3 months prior to treatment	Age, BMI, MVI, Tumor stage	L3 SMI ≤46.4 cm2/m2 for men and 37.6 cm2/m2 for women;
			No-sarcopenia					

Study	Group assignment	patients(M/F)	Age(y)^*^	OS			DFS			Recurrence	OC	MC	Follow-up^†^
				1	3	5	1	3	5				
**Harimoto et al 2013**	Sarcopenia	75(50/25)	67	NR	NR	53	NR	NR	10	NR	24	NR	Every month^#^
	No-sarcopenia	111(95/16)	66	NR	NR	93	NR	NR	37	NR	56	NR	
**Levolger et al 2015**	Sarcopenia	52(39/13)	61(22-86)	37	16	NR	24	8	NR	NR	24	17	>60
	No-sarcopenia	38(24/14)	62(25-77)	35	22	NR	21	9	NR	NR	13	5	
**Valero et al 2015**	Sarcopenia	47	NR	36	29	26	NR	NR	18	NR	19	11	>24
	No-sarcopenia	49	NR	43	35	34	NR	NR	25	NR	9	0	
**Voron et al 2015**	Sarcopenia	59(53/6)	64.55±12.92	41	10	NR	26	10	3	42	23	13	21.2
	No-sarcopenia	50(39/11)	58.25±13.08	48	11	NR	23	9	1	20	18	8	
**Harimoto et al 2016**	Sarcopenia	57(40/17)	76.5±3.9	NR	NR	NR	NR	NR	NR	NR	7	NR	Every month^#^
	No-sarcopenia	82(58/24)	75.9±4.0	NR	NR	NR	NR	NR	NR	NR	15	NR	
**Kamachi et al 2016**	Sarcopenia	61(51/10)	73(49-84)	56	45	31	NR	NR	NR	50	NR	NR	29.7(3.9-107.6)^#^
	No-sarcopenia	31(14/17)	70(47-80)	30	28	21	NR	NR	NR	23	NR	NR	
**Yabusaki et al 2016**	Sarcopenia	89(57/32)	66.2±10.1	71	48	28	NR	NR	NR	NR	18	NR	37.4(1.2-120.7)
	No-sarcopenia	106(100/6)	63.8±10.1	86	55	33	NR	NR	NR	NR	23	NR	
**Takagi et al 2016**	Sarcopenia	118(93/25)	68.6±10	NR	NR	69	NR	NR	NR	NR	NR	19	41.8(1-109)
	No-sarcopenia	136(114/22)	63.1±10.3	NR	NR	112	NR	NR	NR	NR	NR	16	

**Abbreviations:** BMI, body mass index; BSA, body surface area; DFS, disease-free survival; DM, diabetes mellitus; HCC, hepatocellular carcinoma; ICC, intrahepatic cholangio-carcinoma; LT, liver transplantation; MC, major complication; MVI, microvascular invasion; NOS, Newcastle-Ottawa Scale; NR, no report; OC, overall complication; OS, overall survival; PT, prothrombin time; RFA, radiofrequency ablation; TPA, total psoas area;

^*^ Age (year) is given in mean (minimum~maximum) or mean±SD

^^^ Significant clinicopathological factors between patients with and without sarcopenia

^†^Time given in month

^#^all patients were examined monthly for recurrence by ultrasonography and estimation of tumor markers (AFP, DCP) and by CT every 3-6 months.

^&^ The formulae to calculate skeletal muscle area:126.9×BSA-66.2 for men and 125.6×BSA-81.1 for women;

**Table 2:** The Newcastle-Ottawa Scale for assessing the quality of included studies

Included studies		Selection			Comparability		Outcome		Total score
	Representativeness of the exposed cohort	Selection of the non-exposed cohort	Ascertainment of exposure	Demonstration that outcome of interest was not present at start of study	Comparability of cohorts on the basis of the design or analysis	Assessment of outcome	Was follow-up long enough for outcomes to occur	Adequacy of follow up of cohorts	
**Total score**	**1**	**1**	**1**	**1**	**2**	**1**	**1**	**1**	**9**
**Harimoto et al 2013**	1	1	1	1	2	0	0	1	7
**Levolger et al 2015**	1	1	1	1	2	1	1	1	9
**Valero et al 2015**	1	1	1	1	2	1	0	1	8
**Voron et al 2015**	1	1	1	1	2	1	1	1	9
**Harimoto et al 2016**	1	1	1	1	2	1	0	1	8
**Kamachi et al 2016**	1	1	1	1	2	1	0	1	8
**Yabusaki et al 2016**	1	1	1	1	2	1	1	1	9
**Takagi et al 2016**	1	1	1	1	2	1	1	1	9

### Overall survival (OS)

**Figure 2:**
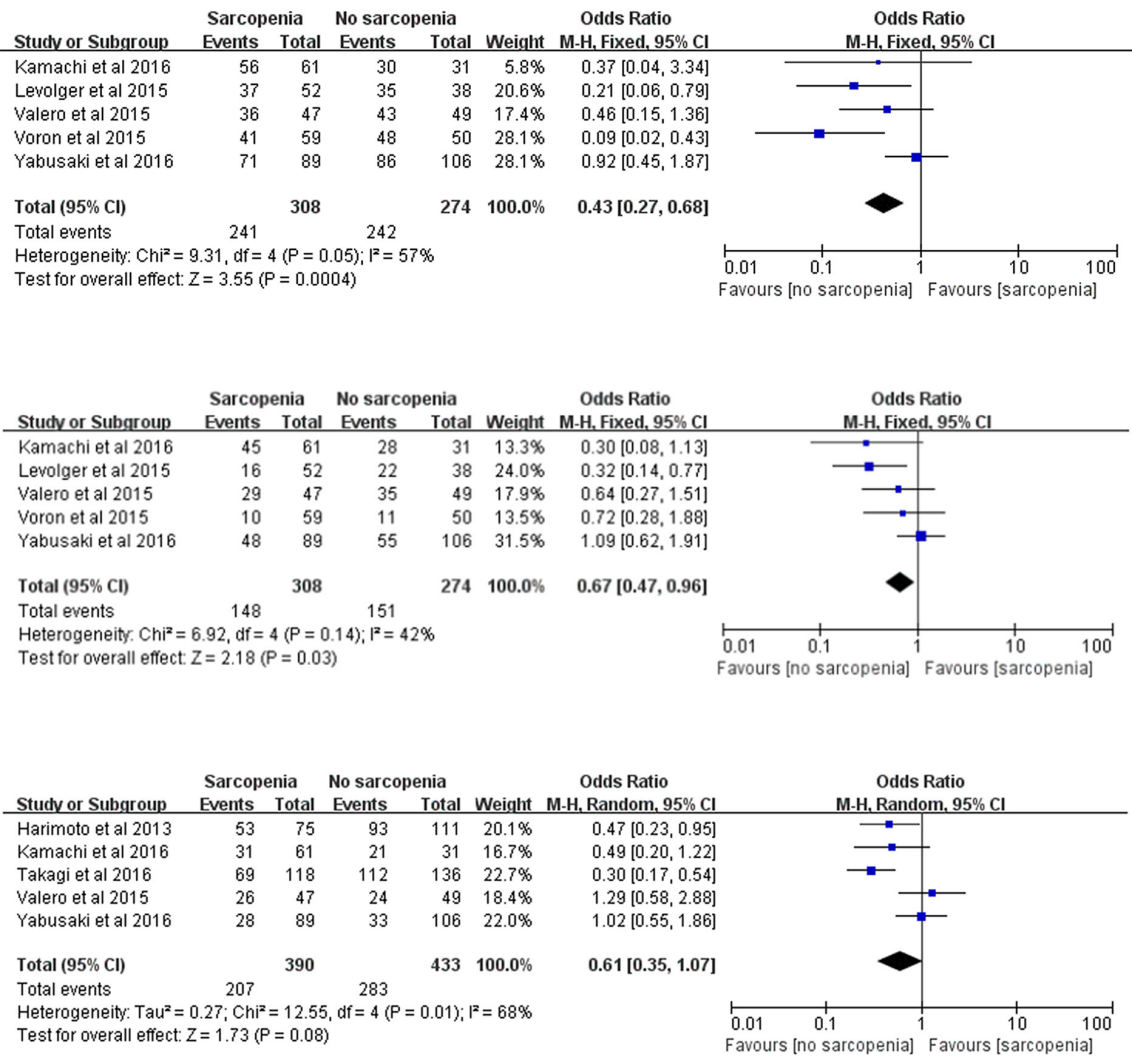
Meta-analysis of the overall 1-, 3- and 5-year survival **(a)** Overall 1-year survival. **(b)** overall 3-year survival. **(c)** overall 5-year survival.

Overall 1-, 3-, and 5-year survival was evaluated in our meta-analysis. Data on overall 1-year survival were available in 5 trials [12, 19, 21–23] including 582 participants, overall 3-year survival was available in 5 trials [12, 19, 21–23] including 582 participants, and overall 5-year survival was available in 5 trials [4, 12, 21, 23, 24] including 823 participants. In our meta-analysis, we concluded that there were significant differences between patients with and without sarcopenia in overall 1- and 3-year survival (1 year: OR: 0.43; 95% CI: 0.27-0.68; *P*=0.0004; 3 year: OR: 0.67; 95% CI: 0.47-0.96; *P*=0.03; Figure 2) with insignificant heterogeneity (1 year: χ^2^=9.31, *P*=0.05, *I^2^*=57%; 3 year: χ^2^=6.92, *P*=0.14, *I^2^*=42%; Figure 2). In contrast, patients with sarcopenia showed an insignificant 39% reduction in OS within 5 years (OR: 0.61; 95% CI: 0.35-1.07; *P*=0.08; Figure 2). A random effects model was used because of statistically significant heterogeneity (χ^2^=12.55, *P*=0.01, *I^2^*=68%; Figure 2).

### Disease-free survival (DFS)

**Figure 3:**
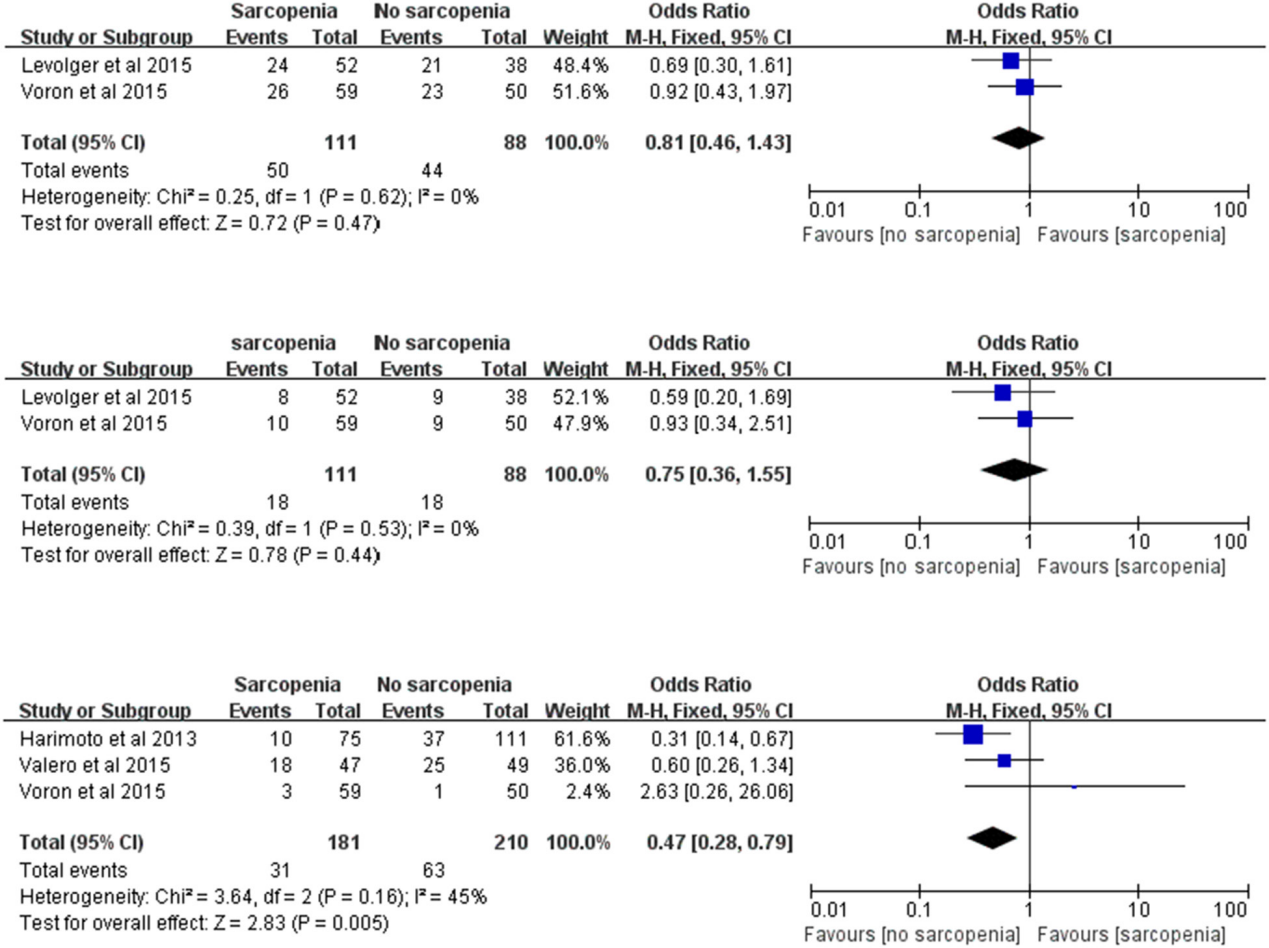
Meta-analysis of the 1-, 3- and 5-year disease-free survival **(a)** 1-year disease-free survival. **(b)** 3-year disease-free survival. **(c)** 5-year disease-free survival.

Overall 1-, 3-, and 5-year DFS was evaluated in our meta-analysis. Two trials [19, 22] including 199 participants reported data on 1-year DFS, two trials [19, 22] including 199 participants reported data on 3-year DFS, and three trials [4, 21, 22] including 391 participants reported data on 5-year DFS. Our meta-analysis revealed that 1- and 3-year DFS showed no significant differences between patients with and without sarcopenia (1 year: OR: 0.81; 95% CI: 0.46-1.43; *P*=0.47; 3 year: OR: 0.75; 95% CI: 0.36-1.55; *P*=0.44; Figure 3). In contrast, patients with sarcopenia showed a significant 53% reduction in DFS within 5 years (OR: 0.47; 95% CI: 0.28-0.79; *P*=0.005; Figure 3). The results were homogeneous (1 year: χ^2^=0.25, *P*=0.62, *I^2^*=0%; 3 year: χ^2^=0.39, *P*=0.53, *I^2^*=0%; 5 year: χ^2^=3.64, *P*=0.16, *I^2^*=45%; Figure 3).

### Recurrence rate

**Figure 4:**
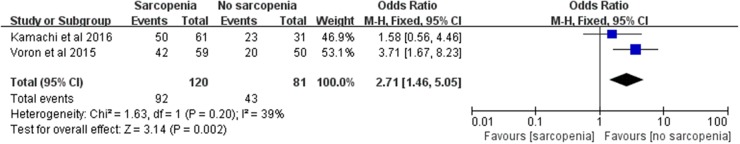
Meta-analysis of the recurrence rate

Two trials [12, 22] including 201 participants reported the recurrence of HCC/ICC after treatment. Our results showed that sarcopenia had a significantly negative impact on patients with HCC/ICC (RR: 2.71; 95% CI: 1.46-5.05; *P*=0.002; Figure 4). The results were homogeneous (χ^2^=1.63, *P*=0.20, *I^2^*=39%; Figure 4).

### Post-treatment complication rate

Complications were evaluated according to the Clavien-Dindo classification, and major complications (MCs) were defined as a Clavien-Dindo grade greater than 3. In our study, the impact of sarcopenia on overall complications (OCs) and major complications was examined.

**Figure 5:**
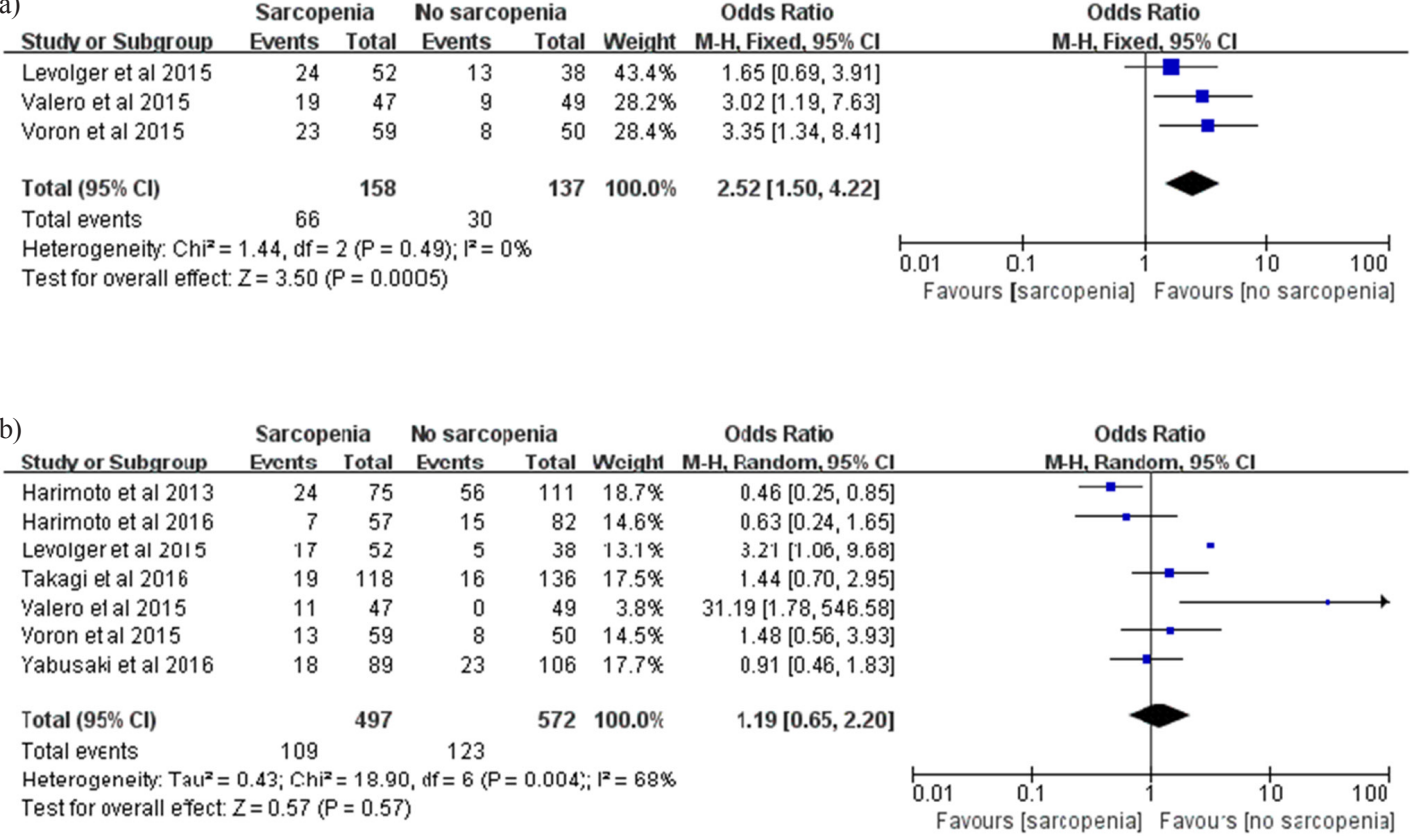
Meta-analysis of the post-treatment complication rate **(a)** Overall complication. **(b)** major complication.

Data on OCs were available in 3 trials [19, 21, 22], including 158 participants who were diagnosed with sarcopenia and 137 participants who were had normal L3 SMI/TPA. In our meta-analysis, we concluded that there were statistically significant differences between the two groups in OC rate (RR: 2.52; 95% CI: 1.50-4.22; *P*=0.0005; Figure 5). The results were homogeneous (χ^2^=1.44, *P*=0.49, *I^2^*=0%; Figure 5).

Data on MCs were available in 7 trials [4, 19, 21–25] among the total of 497 participants who were diagnosed with sarcopenia and 572 participants with normal L3 SMI/TPA. In our meta-analysis, we concluded that there was no statistically significant difference between the two groups in MC rate (RR: 1.19; 95% CI: 0.65-2.20; *P*=0.57; Figure 5). A random effects model was used because of statistically significant heterogeneity (χ^2^=18.90, *P*=0.004, *I^2^*=68%; Figure 5).

## DISCUSSION

Numerous studies have demonstrated that there are correlations between sarcopenia and patients' basic characteristics, such as age [20–22, 24, 26, 27], sex [4, 12, 23, 27], liver function(serum albumin levels) [4, 22, 23], and BMI values [4, 12, 19, 22–24, 26–28]. As reported, sarcopenia occurs during aging, and in patients with primary hepatic malignancies, this is possibly due to cachexia-associated processes. Accordingly, in patients with primary hepatic malignancies, sarcopenia is prevalent across all ages but particularly in the elderly [20–22, 24, 26, 27]. Moreover, because of faster deterioration of muscle mass in men than in women, at older ages, men experience greater losses of muscle mass than women [29, 30]; thus, with regard to sex differences, sarcopenia is more common in men than women [4, 12]. However, a study by Yabusaki et al. [23] showed that the results for sex differences seemed to contradict the present findings. Furthermore, patients with sarcopenia have significantly lower BMI values than patients without sarcopenia, and the serum albumin levels of these patients are generally lower [4, 22, 23]. In limited studies, sarcopenia was correlated with number of tumors [23], micro-vascular invasion [24] and tumor stage [24], which would seem to contradict the present findings. We found no correlations between sarcopenia and tumor size, Child-Pugh grade, Model for End-stage Liver Disease (MELD) score, liver cirrhosis, Barcelona Clinic Liver Cancer (BCLC) classification or tumor differentiation. There have been no reports concerning the relationships between sarcopenia and lymph node metastasis, distant metastasis or positive surgical margin. Additionally, skeletal muscle protein breakdown occurs mainly because of age-related low-grade systemic inflammation, alongside physical inactivity and malnutrition in the elderly. This low-grade systemic inflammation is characterized by elevated pro-inflammatory cytokines and is caused by age-related cell damage and mitochondrial dysfunction [3]. However, there have been no reports concerning the relationship between sarcopenia and white blood cell counts, neutrophil-to-lymphocyte ratios (NLRs) or C-reaction protein (CRP).

Sarcopenia has been revealed to be an unfavorable prognostic factor for clinical outcomes in cancer. However, whether this finding also holds true in patients with primary hepatic malignancies has increasingly been explored recently. Unfortunately, this research field has been severely hampered by a lack of consensus on the definition of sarcopenia, and the definition of sarcopenia has varied even among studies conducted by researchers in the same country [19, 21, 22]. Consequently, the prevalence of sarcopenia has varied widely, from 11.1% to 76% across published studies. CT scanning is the gold standard tool to quantify skeletal muscle mass [31] and hence constitutes a good resource for objective identification of sarcopenia. To avoid intrinsic bias, we chose trials in which sarcopenia was defined using a L3 SMI/TPA cut-off rather than intramuscular adipose tissue content (IMAC) or visceral fat area (VFA) in our meta-analysis. However, the included studies’ evaluations of different cut-off values for sarcopenia undoubtedly led to bias between the associations of sarcopenia with clinical outcomes.

In recent years, the clinical significance of sarcopenia has been reported relatively frequently in patients with primary hepatic malignancies, and concerns about the impact of sarcopenia on outcomes following treatment could not be confirmed in various studies. Levolger et al. [19] showed that sarcopenia was associated with impaired survival (*P*=0.002) in patients with HCC, and MCs were more frequent (*P*=0.033) in sarcopenic patients. However, a study by Yabusaki et al. [23] showed that there was no significant correlation between OS (*P*=0.72), and there was no difference in the incidence of postoperative MCs (*P*=0.62) between the two groups. The difference might have derived from the inclusion criteria because Levolger et al. included patients with hepatocellular carcinoma undergoing treatment with curative intent (hepatectomy or RFA), while Yabusaki et al. enrolled patients who underwent primary hepatectomy for hepatocellular carcinoma. Also, the relatively small number of participants could have biased the results. Our systematic review and meta-analysis confirmed that patients with sarcopenia had a statistically significantly poorer prognosis (overall 1- and 3-year survival, 5-year DFS, incidence of recurrence and post-treatment OCs) than those without sarcopenia. In contrast, there were no significant differences in overall 5-year survival, 1- and 3-year DFS or post-treatment MC.

Yabusaki et al. [23] reported that sarcopenia impairs OS, mainly due to an increase in treatment-related deaths (*P*=0.029). Evidence has shown that sarcopenic patients with primary hepatic malignancies seem to be less able to recover from treatment. Although the mechanisms by which sarcopenia affects patients’ recovery are not fully understood, differences in the severity of the underlying liver disorders impacting skeletal muscle mass might very well play a part. Studies have shown that patients with sarcopenia are more susceptible to bacterial infection, slow wound healing and longer length of hospital stays [32, 33], which might explain why sarcopenia has an adverse impact on short-term overall survival, consistent with our study that patients with sarcopenia had poorer overall 1- and 3-year survival but better overall 5-year survival. However, the available data from our meta-analysis suggested that sarcopenia increases the incidence of recurrence significantly with poor 5-year DFS. As reported, recurrence of HCC after curative treatment remains a major challenge for the management of liver malignancy, and recent studies have widely shown that sarcopenia was a strong and independent prognostic factor for recurrence after liver resection for HCC [22, 34], which was in agreement with our finding. Therefore, whether the reduction in OS found in patients with sarcopenia is due to increased recurrent malignancy or due to increased treatment-related mortality remains controversial. Combining the evidence revealed by Yabusaki et al. [23] that sarcopenia impairs OS mainly due to an increase in treatment-related deaths, we can conclude that sarcopenia impairs OS due to a combination of treatment-related deaths and recurrence of malignancy. That is, treatment-related death plays a primary role in the early phase (within 3 years) post-treatment, while recurrence plays a primary role over the longer term (perhaps more than 5 years) after treatment, which could explain our results that sarcopenia resulted in poorer 5-year DFS but better 1- and 3-year DFS. The reduction in OS (1- and 3-year) in patients with sarcopenia and lack of associations with DFS (1- and 3-year) between groups also suggested that there are perhaps other factors associated with poor survival in patients with sarcopenia. As reported, sarcopenia can be merely a secondary finding of longstanding chronic disease such as diabetes, gastrointestinal disorders, and cardiovascular diseases. This finding is in agreement with our results that sarcopenia was not associated with DFS (1- and 3-year) between groups because non-liver- or non-HCC/ICC-related factors are also related to prognosis. Interestingly, we noted that patients with sarcopenia had poorer 5-year DFS but better overall 5-year survival. The reason for this inconsistency is undoubtedly multi-factorial. As in our previous descriptions, sarcopenia increases the incidence of recurrence significantly, so more patients die from recurrence of malignancy in patients with sarcopenia on the basis of similar OS to that of patients without sarcopenia, which results in poorer 5-year DFS in patients with sarcopenia. Also, the available data suggested that the number of patients/trials on DFS and recurrence might have been too small, so bias cannot be excluded.

Regarding post-treatment complications, they remain controversial. Valero et al. [21] showed that the presence of sarcopenia was an independent predictive factor of OCs and MCs. However, evidence [19] has also shown sarcopenia did not increase the incidence of OCs. The available data from our meta-analysis suggested that sarcopenia increased the incidence of OCs significantly. However, our study provided no evidence of an increased risk of MCs in sarcopenic patients. The reasons below might account for the non-statistically significant difference in MCs: 1) patients with sarcopenia might receive more intensive care before, during and after treatment; and 2) the absence of a sub-analysis of each complication might have caused potential bias because each complication that develops after HCC/ICC treatment could have a different relationship or causality with sarcopenia and a different impact of prognosis.

Our present meta-analysis had several limitations. In particular, the absence of randomized, controlled clinical trials and the preponderance of retrospective studies could have biased the results to some extent. Second, the relatively small number of participants could have biased the results. Third, several trials were excluded because sarcopenia was not evaluated by L3 SMI/TPA, which could have caused potential bias. Fourth, no subgroup analysis could be conducted because the data extracted from the included studies were not disaggregated by groups. Fifth, the absence of a consistent duration between CT scans and treatment might have created potential bias. Sixth, we failed to elucidate whether sarcopenia was truly a causal factor of poor prognosis or merely a concomitant finding of primary hepatic malignancies or a type of co-morbidity.

## MATERIALS AND METHODS

### Search strategy and study selection

This systematic literature review and meta-analysis were performed using the methodology suggested by the PRISMA guidelines. A systematic literature search was performed in English through February 1, 2017, in the following databases: Medline, PubMed, Medline, Embase, Web of Science and the Cochrane Library. The key words used were “sarcopenia,” “hepatocellular carcinoma,” “intrahepatic cholangio-carcinoma,” “primary hepatic malignancy,” “third lumbar skeletal muscle index,” “third lumbar total psoas area,” and abbreviations thereof. The key words were combined with appropriate Boolean operators, and for further relevant articles, we also checked the reference lists of all the identified trials. After completing the literature searches, titles and abstracts of the studies were screened by two authors, and any disagreement was resolved by discussion or, if necessary, adjudicated by a third author.

### Inclusion and exclusion criteria

The inclusion criteria were as follows: 1) a prospective or retrospective cohort study was adopted; 2) the L3 SMI/TPA was measured by CT scan; and 3) patients were examined regularly and had a minimum follow-up duration of 12 months. When the same patient cohort overlapped among different publications, only the latest or complete study was considered.

The following studies were considered to be ineligible: 1) studies associated with animals; 2) case reports or studies in which the control groups were not well designed; 3) post-treatment sarcopenia; 4) and sarcopenia evaluated by intramuscular adipose tissue content (IMAC)/visceral fat area (VFA) rather than by L3 SMI/TPA.

### Data extraction and study quality

Data extraction was performed independently by two authors using a standard form. The following data were extracted from each study: the basic information of the study (surname of the first author and year of publication, country of the procedure performed, study design, group assignment, histopathological type, treatment, CT scan parameters, definition of sarcopenia, number of patients and ages, follow-up duration) and the clinical significance of sarcopenia in the treatment of HCC/ICC (overall survival, disease-free survival, recurrence of malignancy, overall complications and major complications). The methodological quality of each trial was assessed according to the NOS, which evaluates included studies based on three broad perspectives: 1) selection; 2) comparability; and 3) outcome. The NOS assigns a maximum score of 4, 2 and 3 for selection, comparability and outcome respectively. High-quality trials scored ≥7, and moderate-quality trials scored ≥5 (maximum possible score equal to 9).

### Statistical analysis

The statistical analysis was performed independently by two authors according to recommendations from the PRISMA statement and the Cochrane handbook from the Cochrane Collaboration. Pooled relative risk (RR) with 95% confidence intervals (95% CIs) were calculated for each principal dichotomous variable outcome using either a fixed effects model or a random effects model. Values <1 did not favor the sarcopenia group and values >1 favored the sarcopenia group for overall survival and disease-free survival rate (it should have been the opposite for the parameters such as recurrence and complications rate). We analyzed heterogeneity among the studies using Cochrane's Q test and by calculating *I^2^*, with *P* < 0.05 used to denote statistical significance and with *I^2^* calculated to measure the proportion of total variation in the estimates of treatment effect due to heterogeneity beyond chance. No subgroup analysis could be conducted in our meta-analysis because the data extracted from the included studies were not disaggregated by groups. All the statistical analyses were performed using RevMan software, version 5.3, provided by Cochrane Collaboration.

## CONCLUSION

In conclusion, sarcopenia seemed to have a negative effect on OS in patients with primary hepatic malignancies in the early phase post-treatment, but further research is needed to investigate the prognostic impact on OS over the longer term. Also, we concluded that sarcopenia could significantly increase the incidence rates of post-treatment recurrence and overall complications in patients with primary hepatic malignancies. However, due to a lack of the subgroup analysis, sufficient RCTs and prospective cohort studies, the prognostic impact of sarcopenia remains contentious; therefore, future investigations are needed to evaluate the prognostic impact of sarcopenia in primary hepatic malignancies.

## Abbreviations

COPD: chronic obstructive pulmonary disease
DFS: disease-free survival
DM: diabetes mellitus
HCC: Hepatocellular Carcinoma
ICC: intrahepatic cholangio-carcinoma
IMAC: Intramuscular adipose tissue content
L3 SMI: L3 skeletal muscle index
LT: liver transplantation
MC: major complication
NOS: Newcastle-Ottawa Scale
NR: no report
OC: overall complication
OS: overall survival
PRISMA: Preferred Reporting Items for Systematic Reviews and Meta-Analysis
RFA: radiofrequency ablation
VFA: visceral fat area
